# Why do results conflict regarding the prognostic value of the methylation status in colon cancers? the role of the preservation method

**DOI:** 10.1186/1471-2407-12-12

**Published:** 2012-01-13

**Authors:** Benjamin Tournier, Caroline Chapusot, Emilie Courcet, Laurent Martin, Côme Lepage, Jean Faivre, Françoise Piard

**Affiliations:** 1 Institut National de la Sante et de la Recherche Médicale, Université de Bourgogne, U866 Dijon, France; 2 Service de Pathologie, CHU, Dijon, France; 3 Registre Bourguignon des Cancers Digestifs, CHU, Dijon, France; 4 Centre de ressources biologiques Ferdinand Cabanne, Dijon, France

## Abstract

**Background:**

In colorectal carcinoma, extensive gene promoter hypermethylation is called the CpG island methylator phenotype (CIMP). Explaining why studies on CIMP and survival yield conflicting results is essential. Most experiments to measure DNA methylation rely on the sodium bisulfite conversion of unmethylated cytosines into uracils. No study has evaluated the performance of bisulfite conversion and methylation levels from matched cryo-preserved and Formalin-Fixed Paraffin Embedded (FFPE) samples using pyrosequencing.

**Methods:**

Couples of matched cryo-preserved and FFPE samples from 40 colon adenocarcinomas were analyzed. Rates of bisulfite conversion and levels of methylation of *LINE-1, MLH1 *and *MGMT *markers were measured.

**Results:**

For the reproducibility of bisulfite conversion, the mean of bisulfite-to-bisulfite standard deviation (SD) was 1.3%. The mean of run-to-run SD of PCR/pyrosequencing was 0.9%. Of the 40 DNA couples, only 67.5%, 55.0%, and 57.5% of FFPE DNA were interpretable for *LINE-1, MLH1*, and *MGMT *markers, respectively, after the first analysis. On frozen samples the proportion of well converted samples was 95.0%, 97.4% and 87.2% respectively. For DNA showing a total bisulfite conversion, 8 couples (27.6%) for *LINE-1*, 4 couples (15.4%) for *MLH1 *and 8 couples (25.8%) for *MGMT *displayed significant differences in methylation levels.

**Conclusions:**

Frozen samples gave reproducible results for bisulfite conversion and reliable methylation levels. FFPE samples gave unsatisfactory and non reproducible bisulfite conversions leading to random results for methylation levels. The use of FFPE collections to assess DNA methylation by bisulfite methods must not be recommended. This can partly explain the conflicting results on the prognosis of CIMP colon cancers.

## Background

Epigenetic dysregulation is a major event in the origin of many cancers [1]. DNA methylation, the most widely studied epigenetic mechanism, occurs in cytosines that precede guanines (CpG dinucleotides). The CpG dinucleotides may be found concentrated in regions called CpG islands, commonly located in gene promoters. In colon cancers, a number of tumour suppressor genes are transcriptionally silenced by promoter CpG island hypermethylation [2,3]. Among them, one subset referred to as the CpG island methylator phenotype (CIMP) exhibits widespread promoter methylation [2,4]. Studies on CIMP status and survival in colon cancers have yielded somewhat inconsistent results [5-13]. One of our previous studies [5] as well as other studies [8,11,13] suggested that the CIMP had an adverse effect on survival in MSS (Microsatellite Stable) tumours, while in other reports CIMP-H (CIMP-High) status was independently associated with low specific mortality [9,12]. These discrepancies might result from differences in the choice of tissue samples. The quality of DNA samples depends especially on the material available which may be cryo-preserved or formalin-fixed paraffin embedded (FFPE) tissue. The cryo-preservation technique provides the best protection of the DNA, but these specimens are far less common than FFPE tissue from pathology departments, which is a vast resource. The quality of archived specimens (FFPE tissues) depends on the fixation and storage conditions employed and can vary greatly from one sample to another. Moreover, formalin fixation induces degradation and cross-linkings between proteins and proteins/DNA bases. FFPE tissues thus produce a poorer yield of DNA. To evaluate DNA methylation, the gold-standard method is based on sodium bisulfite conversion [14], in which unmethylated cytosines are converted into uracils. Then, after PCR, it is possible to differentiate unmethylated cytosines (replaced by thymines) from methylated cytosines which are protected from bisulfite conversion [15]. However, the DNA bisulfite conversion step is a chemical reaction that also degrades DNA. All things considered, cross-linkings and DNA degradation caused by fixation, extraction and conversion methods have a negative impact on conversion efficacy, while DNA conversion must be total and reproducible to allow meaningful interpretation of results.

A number of techniques have been employed to analyze converted DNA [16-19]. These include MSP (methylation-specific polymerase chain reaction), Methylight (real time PCR), SMART-MSP (Sensitive Melting Analysis after Real Time PCR - methylation-specific polymerase chain reaction), MS-HRM (Methylation Sensitive - High Resolution Melting) and pyrosequencing. All these techniques are based on DNA bisulfite conversion. Among them, pyrosequencing is the only one that comprises an in-built measure to check the completeness of bisulfite conversion (conversion control) which allows a precise evaluation of the quality of the conversion. Moreover, it gives the percentage of methylated allele for each CpG dinucleotide analysed.

It is important to investigate why there are discrepancies in the prognostic value of the CIMP phenotype in colorectal cancers between studies especially as few explanations have been proposed. The aim of this study was to evaluate the feasibility of analyzing DNA methylation from DNA extracted from FFPE tissues and to compare the results with those obtained from frozen material using pyrosequencing technology. To this end, three different known markers of methylation (*LINE-1, MLH1 *and *MGMT*) were chosen. Results of this work could help establish a standard method for assessing DNA methylation and thus make it possible to compare results obtained in this field.

## Methods

### Samples

Forty tumour tissue samples from patients resected for a colon adenocarcinoma were included. For each tumour, one frozen sample previously stored in the Ferdinand Cabanne Biological Resources Centre (Dijon, France) and one FFPE (Formalin Fixed Paraffin Embedded) tissue block, were available. The CPP EST I committee (Comité de Protection des Personnes: Protection of Individuals committee) approved the use of these biological collections. The tissue samples were considered surgical waste in accordance with French ethical laws (L.1211-3 to L.1211-9).

Quality control of frozen samples was carried out before DNA extraction according to a strict process. Three sections, each separated by 15 other sections, were cut on a cryostat and stained with Hematein-Eosin-Safran (HES). These three stained sections were analyzed by a pathologist and the proportion of tumour tissue was recorded. The sections retained for the study were the consecutive sections located between two stained sections containing at least 40% of tumour cells. The choice of the percentage of tumor cellularity was established in our laboratory from previous analyses (data not shown). This percentage was ample for methylation quantification.

For FFPE samples, the slides were also reviewed by a pathologist in order to select an area rich in tumour cells (> 40% of tumour cells). The initial paraffin blocks were manually dissected and the selected tumour area was embedded in a new paraffin block.

For all pairs of FFPE/frozen samples, we always checked that the difference in tumour cellularity between selected sections was slight in order to be able to compare both tissues.

### DNA extraction from frozen tissues: Nucleospin^®^96 tissue kit (Macherey Nagel^®^)

Twenty 50 μm-tissue sections were crushed with two stainless steel balls in a mixture containing RLT buffer (Qiagen^®^) and 1% β-mercapto-ethanol (14.3 M; Sigma Aldrich^®^). Half of each sample was then centrifuged for 10 min (10000 rcf (relative centrifugal force)). The pellets were suspended in 180 μl of lysis buffer (T1 buffer) and 25 μl proteinase K. Samples were kept at 56°C overnight. DNA extraction was performed with a TECAN^® ^automate following the supplier's recommendations. DNA was released in 100 μl elution buffer.

### DNA extraction from formalin-fixed paraffin embedded (FFPE) tissues

#### Classical method

For each sample, ten 15 μm-thick tissue sections underwent proteinase K digestion (> 600 mAU/ml, Qiagen^®^) at 56°C for one night. DNA was extracted using a Bionobis kit (Magtration^®^-Magazorb^®^) and a Bionobis^® ^automat according to the manufacturer's instructions. The samples were lysed, washed and then adsorbed onto magnetic silicate particles. DNA was immobilized by magnetic attraction, washed again and released in 100 μl of water. DNA extracted with this technique was analyzed for the *LINE-1 *marker.

#### Dedicated Method to FFPE tissue: QIAamp^® ^DNA FFPE Tissue kit (QIAGEN^®^)

For each sample, ten 15-μm-thick tissue sections were transferred into a 1.5 ml tube. The extraction was performed according to the manufacturer's protocol. For further details see Additional file 1. In brief, tissue sections were first dewaxed using toluene and lysed under denaturing conditions with proteinase K. Then lysates were incubated at 90°C. DNA was bound to the membrane and contaminants were washed away by several washing steps. Finally pure DNA was eluted.

### Quantification and quality assessment of DNA

DNA was quantified with a Nanodrop^® ^spectrophotometer (Thermo scientific^®^) and diluted at 50 ng/μl. The quality of the DNA was assessed by multiplex PCR which amplified microsatellite regions as previously described [20,21].

### DNA bisulfite conversion - EpiTect Bisulfite kit (QIAGEN^®^)

For bisulfite conversion, the optimal quantity of DNA was determined in a preliminary work (data not shown). Five hundred nanograms of genomic DNA of each sample were used and bisulfite treatment was performed according to the manufacturer's protocol. For further details see Additional file 1. In brief, first the bisulfite mediated conversion of unmethylated cytosines was performed. After, the converted single strand DNA was bound to the membrane of the EpiTect spin columns. Membrane bound DNA was washed, then desulfonated, and washed again to remove the desulfonation agent. Finally pure converted DNA was eluted.

### PCR and pyrosequencing assays

We previously optimized PCR conditions for each amplification by testing the following conditions: annealing temperature, magnesium concentration and cycle number.

#### Measurement of LINE-1 methylation level

*LINE-1 *PCR amplifies a 154 base-pair sequence in the consensus promoter of *LINE-1 *elements (acc. n° X58075). It was performed using custom primers (Additional file 2), designed with the PyroMark assay design 2.0 (Qiagen^®^). The PCR reaction was carried out in a 50 μL final volume comprising 25 μL of PyroMark Master Mix (containing PCR buffer, dNTP and HotStar Taq DNA polymerase), 5 μL of Coral Load buffer, 4 μL of 25 mM MgCl_2_, 1 μL of the forward and biotinylated reverse primers (0.2 μM final concentration), 13 μL of RNase free water and 1 μL of bisulfite treated DNA (Qiagen^®^). PCR cycling conditions were as follows: initial denaturing at 95°C for 15 min, 45 cycles of 95°C for 30 s, 56°C for 30 s, and 72°C for 30 s and final extension at 72°C for 10 min. Reverse single-stranded biotinylated templates were isolated using the PyroMark Vacuum Prep WorkStation (Qiagen^®^). Forty-six micro-litres of PCR product were added to 38 μL of binding buffer (Qiagen^®^) and 2 μL streptavidin sepharose high-performance beads (GE Healthcare^®^). The mixtures were shaken for 10 min at 1400 rpm (revolution per minute). After agitation, beads covered by biotinylated DNA were collected and retained on filter probes by permanent vacuum. The filter probes were successively immerged in different baths: in ethanol 70% for 5 s, in PyroMark denaturation solution for 5 s and in PyroMark wash buffer 1× for 15 s (Qiagen^®^). Then the vacuum was turned off and the beads fixing DNA strands were released into a 24 well plate containing 25 μl of annealing buffer with 0.3 μM of sequencing primer in each well. The sequencing plate was kept at 80°C for 2 min and at room temperature for 5 min. Pyrosequencing reactions were performed in a PyroMark Q24 MDx system using PyroGold reagents (Qiagen^®^). The nucleotide dispensation order used is indicated in Additional file 2. Results were analyzed using PyroMark Q24 2.0.6 Software. To ensure successful bisulfite conversion of unmethylated cytosines, an internal conversion control that corresponded to the position of a non-CG cytosine (not subject to methylation) was present in the dispensation sequence. The average *LINE-1 *methylation level was calculated as the mean of the proportions of C (%) at the 3 CpG sites analysed, which were located at positions +319, +322 and +329 (positions of the corresponding Guanine in the forward DNA strand, in relation to the first nucleotide base of the consensus promoter sequence) and this indicated the level of methylation of *LINE-1 *elements.

#### Measurement of MLH1 and MGMT methylation levels

To investigate the methylation level of *MLH1 *and *MGMT *genes, we used the PyroMark Q24 kits (Qiagen^®^) as we did not succeed in designing amplicons targeting the same CpG sites with a smaller length than those proposed in these kits. The *MLH1 *kit was designed to detect the methylation level in a region -209 to -181 from the transcription start site of the *MLH1 *gene and the *MGMT *kit to detect the methylation level in a region +17 to +39 in exon 1 of the *MGMT *gene. PCR reactions were performed according to the manufacturer's instructions. Then, 22 μL of PCR product was added to 40 μL of binding buffer, 16 μL of ultrapure water and 2 μL of streptavidin sepharose high-performance beads. The single-stranded biotinylated templates were purified similarly to the *LINE-1 *assay (mentioned above). The sequencing plate containing purified DNA strands and sequencing primer was kept at 80°C for 2 min and at room temperature for 5 min. Pyrosequencing reactions were performed in a PyroMark Q24 MDx system using PyroGold reagents (Qiagen^®^). The nucleotide dispensation orders of *MLH1 *and *MGMT *assays are indicated in Additional file 2. Results were analyzed using PyroMark Q24 2.0.6 Software. To ensure successful bisulfite conversion of unmethylated cytosines, an internal conversion control that corresponded to the position of a non-CG cytosine (not subject to methylation) was present in the dispensation sequence. The average methylation level for the two markers was calculated as the mean of the proportions of C (%) at the 5 CpG sites that were analysed.

### Assessment of reproducibility of bisulfite conversion, PCR/Pyrosequencing

**Figure 1:**
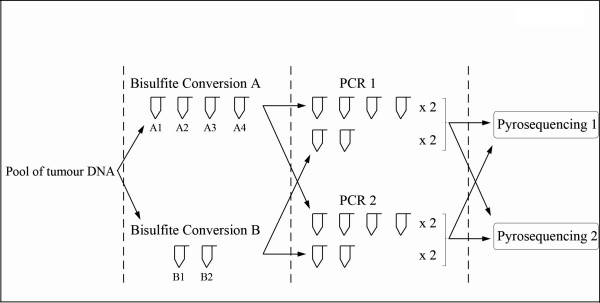
**Schematic representation of the procedure for evaluating methylation variability, by pyrosequencing, from cryo-preserved tissue DNA**. A1, A2, A3 and A4 symbols were the four replicates of the bisulfite conversion A of the pool of tumour DNA performed on day 1, and B1 and B2 the two replicates of the bisulfite conversion B performed on day 2. PCR 1 and 2 were two similar but independent PCRs performed one day apart in duplicate. Pyrosequencing 1 and 2 were two similar but independent pyrosequencing procedures performed one day apart.

The reproducibility of bisulfite conversion efficiency and PCR/Pyrosequencing and the variability of methylation measurement generated using the same frozen DNA sample were evaluated as shown in Figure 1. In practice, a pool of tumour DNA from cryo-preserved tissues was used; identical bisulfite conversions were performed one day apart (day 1: conversion A, day 2: conversion B). Four conversions were carried out on day 1 (A1, A2, A3, A4) and two on day 2 (B1 and B2). Then, two independent PCRs (one day apart) were carried out in duplicate from each converted sample and then two independent pyrosequencing procedures, one day apart, were also performed. We measured the level of *LINE-1 *methylation for the three sites by pyrosequencing on each of these 24 PCR products [six converted DNA samples × two PCR × 2 (in duplicate)]. For each case and each site we measured the standard deviations (SD) on A1 through B2 of the four measures of levels of methylation, which could primarily depend on variations in bisulfite conversion. The repetition of PCR and Pyrosequencing allowed us to determine the exact levels of methylation on the set of bisulfite-converted DNA samples. The SD calculated between the different measures of methylation of each site for each given converted sample could primarily depend on day-to-day variations in the pyrosequencing assay. In addition, the maximum variation in the methylation level in absolute value was calculated for each site.

## Results

### Variability of measurement of the methylation level of DNA from cryo-preserved tissue

Data for *LINE-1 *methylation levels of the pool of tumour DNA sample for the three CpG sites analyzed are summarized in Table 1. The mean of SDs observed after bisulfite treatments (bisulfite-to-bisulfite SD) was 1.3% and the mean of SDs observed after PCR/pyrosequencing was 0.9%. The variability of measurement of the methylation level induced by bisulfite conversion and PCR/pyrosequencing was 1.4%. for site 1, 1.0% for site 2 and 1.4% for site 3.

**Table 1:** Variability of *LINE-1 *methylation levels of DNA from cryo-preserved tissue

converted DNA samples	PCR 1 (% of methylation)						PCR 2 (% of methylation)						SD due to different PCR and different pyrosequencing			
	pyrosequencing 1			pyrosequencing 2			pyrosequencing 1			pyrosequencing 2						
	CpG site 1	CpG site 2	CpG site 3	CpG site 1	CpG site 2	CpG site 3	CpG site 1	CpG site 2	CpG site 3	CpG site 1	CpG site 2	CpG site 3	CpG site 1	CpG site 2	CpG site 3	
A1	55.9	62.9	62.3	55.0	62.1	62.2	56.5	61.6	59.3	54.6	63.4	61.4	0.9	0.8	1.4	mean = 0.9
A2	***53.1***	63.1	59.7	56.0	63.3	59.5	53.2	62.7	60.0	57.5	62.3	60.5	2.2	0.4	0.4	
A3	55.7	61.9	***58.6***	55.4	61.8	61.1	54.8	***60.6***	59.6	55.3	62.5	61.6	0.4	0.8	1.4	
A4	54.1	63.8	60.7	56.4	63.6	59.9	56.4	63.7	60.3	55.8	64.1	59.6	1.1	0.2	0.5	
B1	56.3	61.8	62.7	57.8	63.1	**63.5**	58.0	62.9	60.6	56.7	64.1	61.4	0.8	0.9	1.3	
B2	55.9	63.4	62.9	57.9	64.0	63.2	55.2	63.1	62.5	**58.6**	**64.7**	63.3	1.6	0.7	0.4	
bisulfite-to-bisulfite SD	1.3	0.8	1.8	1.2	0.9	1.7	1.7	1.1	1.1	1.5	0.9	1.2				
	mean = 1.3															

SD between all measures of site 1																1.4%

SD between all measures of site 2																1.0%

SD between all measures of site 3																1.4%

The levels of methylation (%) of the three studied CpG sites are given according to the converted DNA sample analysed (A1 through B2) and for each condition of analysis (PCR and pyrosequencing). Numbers in bold italics indicate minimal values of methylation on all measures for each CpG site. Numbers in bold indicate maximal values of methylation on all measures for each CpG site

### Qualitative analysis of bisulfite conversion for FFPE tissues

#### Classical extraction method

For DNA extracted with the classical method, the bisulfite conversion was tested for the *LINE-1 *marker. Twenty five DNA samples displayed an uncertain ("check": 5.0 to 7.0% of unconverted cytosines) or poor conversion ("failed": more than 7.0% of unconverted cytosines) in the first analysis and 24 in the second analysis. The results of the control of conversion were not always similar in the two analyses. Cases n° 2, 5, 6, 9, 14, 27 and 37 showed invalid control of conversion ("check" or "failed") during the first analysis and valid control ("passed": less than 5.0% of unconverted cytosines) of conversion during the second analysis. On the other hand, six cases (n° 8, 18, 20, 22, 23) showed valid conversion after the first assay and invalid control of conversion at the second analysis. For nine cases (n° 3, 16, 24, 28, 30, 32, 33, 36, 38) the controls showed valid conversion for the two analyses. By combining the valid results of the two analyses, 55.0% of the cases (22 cases) were interpretable.

#### Dedicated extraction kit for the LINE-1 marker

All the DNAs could be amplified. The internal controls showed valid conversion for 27 cases (67.5%) in the first analysis and 28 cases (70.0%) after the second analysis with the *LINE-1 *marker. Cases n° 2, 11 and 19 showed invalid control of conversion ("check" or "failed") in the first analysis and valid control of conversion ("passed") in the second analysis. On the other hand, two cases (n° 4, 39) showed successful conversion after the first assay and an invalid control of conversion in the second analysis. By combining the valid results of the two analyses, 75.0% of the cases were interpretable (30 of the 40 FFPE DNAs).

#### Dedicated extraction kit for MLH1 and MGMT

With the *MLH1 *marker, 11 (27.5%) DNAs could not be amplified. Among those that were amplified (29/40), controls showed valid conversion for 23 cases (57.5% of the 40 cases) in the first assay and 27 after combining the two analyses. Concerning the *MGMT *marker, all the DNAs could be amplified. The internal controls of conversion were valid for 22 cases (55.0%) in the first analysis. Among the 18 cases which gave an invalid control of conversion, 13 (n° 4, 7, 17, 19, 20, 22, 26, 30, 33, 34, 35, 39 and 40) gave a valid control of conversion in the second analysis. By combining the valid results, 35 of the 40 (87.5%) FFPE DNAs showed valid control of conversion.

### Qualitative analysis of bisulfite conversion for cryo-preserved tissues

The results of bisulfite conversion for the three markers after two analyses are given in Table 2. Assessment of the *LINE-1 *marker showed that two frozen samples failed to be converted (cases 19 and 27) in the first assay (95.0% of well converted DNA) and one was not amplified after the two analyses (case 27). For the *MLH1 *marker, in the first assay, only one DNA (n° 21) had an invalid control of conversion, the other 38 DNAs (97.4%) displayed satisfactory bisulfite conversion (DNA n°27 was not amplified). The results were similar after the second assay. For the *MGMT *marker, in the first analysis, bisulfite conversion was not successful for five cases (n°7, 19, 26, 37 and 38) and DNA n°27 was not amplified. So 87.2% of cases showed valid controls of conversion. The same results were obtained in the second analysis.

**Table 2:** Results of internal controls of conversion of the three markers

Identity	**FFPE DNA **1		**FFPE DNA **2		**FFPE DNA **2		**FFPE DNA **2		Cryo. DNA		Cryo. DNA		Cryo. DNA	
	
	*LINE-1*		*LINE-1*		*MLH1*		*MGMT*		*LINE-1*		*MLH1*		*MGMT*	
	
	PCR 1	PCR 2	PCR 1	PCR 2	PCR 1	PCR 2	PCR 1	PCR 2	PCR 1	PCR 2	PCR 1	PCR 2	PCR 1	PCR 2
1	**check**	**failed**	passed	passed	passed		passed		passed	passed	passed		passed	

2	**failed**	passed	**check**	passed	**⁄**		passed		passed	passed	passed		passed	

3	passed	passed	**check**	**check**	**⁄**		**failed**	**failed**	passed	passed	passed		passed	

4	**failed**	**failed**	passed	**check**	**⁄**		**failed**	passed	passed	passed	passed		passed	

5	**failed**	passed	**failed**	**check**	**⁄**	passed	passed	passed	passed	passed	passed		passed	

6	**check**	passed	passed	passed	passed		passed		passed	passed	passed		passed	

7	**failed**	**failed**	**failed**	**check**	passed	passed	**failed**	passed	passed	passed	passed		**check**	**check**

8	passed	**failed**	passed	passed	passed		passed		passed	passed	passed		passed	

9	**check**	passed	passed	passed	passed		passed		passed	passed	passed		passed	

10	**failed**	**failed**	passed	passed	passed		passed		passed	passed	passed		passed	

11	**failed**	**failed**	**check**	passed	**⁄**	passed	**check**	**check**	passed	passed	passed		passed	

12	**failed**	**failed**	**failed**	**check**	passed	**⁄**	passed	**check**	passed	passed	passed		passed	

13	**check**	**check**	passed	passed	passed		passed		passed	passed	passed		passed	

14	**check**	passed	passed	passed	**⁄**	passed	passed		passed	passed	passed		passed	

15	**check**	**check**	passed	passed	passed		passed		passed	passed	passed		passed	

16	passed	passed	**check**	**check**	**⁄**	passed	passed	passed	passed	passed	passed		passed	

17	**failed**	**failed**	passed	passed	passed		**check**	passed	passed	passed	passed		passed	

18	passed	**failed**	passed	passed	**⁄**		passed		passed	passed	passed		passed	

19	**check**	**failed**	**failed**	passed	**⁄**		**check**	passed	**check**	passed	passed		**failed**	**check**

20	passed	**check**	passed	passed	passed		**failed**	passed	passed	passed	passed		passed	

21	**check**	**check**	**failed**	**check**	**⁄**		**failed**	**failed**	passed	passed	**check**	**failed**	passed	

22	passed	**check**	**check**	**check**	passed		**failed**	passed	passed	passed	passed		passed	

23	passed	**check**	passed	passed	passed		passed		passed	passed	passed		passed	

24	passed	passed	passed	passed	passed		passed		passed	passed	passed		passed	

25	passed	**failed**	passed	passed	**⁄**		passed	passed	passed	passed	passed		passed	

26	**check**	**check**	passed	passed	passed		**check**	passed	passed	passed	passed		**check**	**check**

27	**check**	passed	passed	passed	passed		passed		**failed**	**⁄**	**⁄**		**⁄**	

28	passed	passed	passed	passed	passed		**failed**	**failed**	passed	passed	passed		passed	

29	**check**	**check**	passed	passed	passed		passed		passed	passed	passed		passed	

30	passed	passed	passed	passed	**failed**	**check**	**check**	passed	passed	passed	passed		passed	

31	**failed**	**failed**	passed	passed	**⁄**		passed		passed	passed	passed		passed	

32	passed	passed	passed	passed	passed		passed		passed	passed	passed		passed	

33	passed	passed	**check**	**check**	passed	**⁄**	**check**	passed	passed	passed	passed		passed	

34	**check**	**check**	passed	passed	**⁄**		**failed**	passed	passed	passed	passed		passed	

35	**check**	**failed**	passed	passed	**⁄**		**failed**	passed	passed	passed	passed		passed	

36	passed	passed	passed	passed	**failed**	**failed**	passed		passed	passed	passed		passed	

37	**check**	passed	**check**	**failed**	passed	passed	**failed**	**failed**	passed	passed	passed		**check**	**check**

38	passed	passed	**check**	**check**	passed	passed	passed		passed	passed	passed		**failed**	**failed**

39	**check**	**check**	passed	**check**	passed		**check**	passed	passed	passed	passed		passed	

40	**check**	**check**	passed	passed	**⁄**		**failed**	passed	passed	passed	passed		passed	

From left to right the table gives results for DNA from FFPE tissues extracted with the classical method (FFPE DNA 1), extracted with the dedicated method (FFPE DNA 2) and DNA from cryo-preserved tissues (Cryo. DNA). "Passed" indicates a good conversion (0.0 to 5.0% of unconverted cytosines). "Check" indicates an uncertain conversion (5.0 to 7.0% of unconverted cytosines). "Failed" indicates a poor conversion (more than 7.0% of unconverted cytosines). Slash bar indicates there was no amplification. Results in bold emphasize controls of conversion giving "check" or "failed" results.

### Comparison of the controls of conversion obtained for the three markers on the same DNA sample

The results of the conversion controls were not always similar for a given DNA sample according to the marker used. FFPE DNAs n° 5, 7, 12, 16, 22, 37 and 38 showed good control of conversion with the *MLH1 *marker but an uncertain or failed control of conversion with the *LINE-1 *marker. Opposite results were observed for the two FFPE DNAs n°30 and 36. When amplified for the *MGMT *marker, FFPE DNAs n°5, 12, 16, 22, 33 and 38 showed good control of conversion but an uncertain or failed control of conversion with the *LINE-1 *marker. Opposite results were observed for the two FFPE DNAs n°28 and 11. For cryo-preserved tissues, DNA n° 21 displayed a valid control of conversion for *LINE-1 *and *MGMT *markers and an uncertain or failed control of conversion when amplified for the *MLH1 *marker. During *MGMT *amplification, five DNAs (cases n°7, 19, 26, 37, 38) showed uncertain or failed controls of conversion but valid controls of conversion for *LINE-1 *and *MLH1 *markers.

### Quantitative analysis of methylation levels for cryo-preserved and FFPE tissues (DNA extracted with the dedicated kit)

The methylation levels of the 40 couples of DNA extracted from the same sample (cryo-preserved or FFPE) for the three markers are given in Table 3. Considering our previous results on the variability of the methylation level measure of DNA from cryo-preserved tissues for the *LINE-1 *marker (Table 1), we selected the minimal and maximal values of the methylation level for each CpG site. We calculated the difference between the two values to set up a threshold beyond which the difference in the methylation value was not due to the variability of measurements. The differences were 5.6% for CpG site 1, 4.1% for CpG site 2 and 4.9% of methylation for CpG site 3. So we set an arbitrary threshold of 6.0% beyond which the difference was considered significant.

**Table 3:** Differences in methylation levels for each couple of frozen/FFPE DNA according to the three markers

Identity	Levels of *LINE-1 *methylation (% of methylation)		Difference of methylation	Levels of *MLH1 *methylation (% of methylation)		Difference of methylation	Levels of *MGMT *methylation (% of methylation)		Difference of methylation
						
	DNA from cryo-preserved tissues	DNA from FFPE tissues		DNA from cryo-preserved tissues	DNA from FFPE tissues		DNA from cryo-preserved tissues	DNA from FFPE tissues	
1	62.0	66.3	-4.3	51.5	57.7	**-6.2**	1.7	4.3	-2.6

2	66.1	67.2	-1.1	4.8	-	**-**	2.0	6.0	-4.0

3	64.6	64.8 †	-0.2	47.6	-	**-**	57.5	71.9 ‡	**-14.3**

4	57.2	52.3	5.0	6.8	-	**-**	55.8	49.3	**6.5**

5	61.6	64.2 †	-2.6	5.3	1,7*	3.6	1.9	6.2	-4.2

6	47.5	49.2	-1.7	2.9	3.9	-1.0	38.6	46.1	***-7.5***

7	59.4	56.6 †	2.9	5.1	1.9	3.3	59.4 †	43.4	**16.0**

8	47.9	46.5	1.3	3.4	1,5*	1.9	1.7	5.7	-4.0

9	64.5	61.2	3.3	33.1	13.3	**19.7**	14.7	18.2	-3.5

10	61.4	54.1	**7.3**	5.6	1.4	4.2	19.2	32.3	**-13.1**

11	60.5	50.1	**10.4**	3.9	3,4*	0.6	19.8	35.6 †	**-15.8**

12	60.2	62.2 †	-2.0	4.1	2.8	1.3	2.0	5.1	-3.2

13	45.1	62.5	**-17.5**	45.2	2.6	**42.7**	2.1	4.3	-2.2

14	57.0	60.8	-3.8	3.0	7,1*	-4.1	1.4	1.9	-0.5

15	59.2	63.2	-4.0	2.5	5.6	-3.1	1.4	4.9	-3.5

16	52.8	47.5 †	5.2	2.6	3,3*	-0.7	1.6	5.9	-4.3

17	61.0	55.6	5.3	77.7	4.6	**73.1**	1.7	30.2	**-28.5**

18	59.6	53.5	**6.2**	4.8	-	**-**	28.9	25.3	3.6

19	43.3	54.4	**-11.2**	2.2		**-**	73.9 †	62.2	**11.7**

20	61.7	57.6	4.1	4.6	3.5	1.1	15.7	25.2	**-9.5**

21	65.2	66.1 †	-1.0	6.1 †	-	-	2.2	7.0 ‡	-4.8

22	50.9	49.1 †	1.8	3.5	3.3	0.3	1.9	6.8	-5.0

23	61.6	59.4	2.2	3.5	2.1	1.4	1.6	4.6	-2.9

24	59.3	58.0	1.4	2.4	3.5	-1.1	20.8	32.4	**-11.7**

25	61.7	71.0	**-9.3**	4.1	-	**-**	31.7	1,9*	**29.8**

26	56.7	52.2	4.4	2.4	0.9	1.5	52.8 †	20.4	**32.4**

27	45.5 ‡	44.3	1.3	-	5.9	-	-	4.2	**-**

28	65.3	57.4	**7.9**	6.6	2,7*	3.9	41.8	25.2 ‡	**16.6**

29	64.0	57.3	**6.7**	3.4	2,4*	1.0	2.2	3.3	-1.1

30	56.4	56.7	-0.3	11.5	7.3	4.3	3.9	5.3	-1.5

31	62.0	62.9	-1.0	32.8	-	-	5.7	5.3	0.4

32	67.2	62.0	5.2	3.9	5.2	-1.2	19.0	51.8	**-32.8**

33	60.2	62.4 †	-2.3	4.0	3.9	0.1	2.0	7.9	-5.9

34	62.0	61.7	0.3	3.6	-	-	2.2	5.9	-3.7

35	56.9	53.3	3.5	3.8	-	-	2.1	5.6	-3.5

36	40.5	41.1	-0.6	3.0	3.6	-0.6	1.7	5.2	-3.5

37	59.0	62.1 †	-3.0	2.7	3.3	-0.6	88.3 †	73.1 ‡	**15.1**

38	62.3	58.5 †	3.8	4.3	1.5	2.8	68.2	71.7	-3.5

39	50.4	53.2	-2.9	4.8	4.5	0.3	1.9	4.6	-2.7

40	36.2	34.7	1.5	3.1	-	**-**	2.0	5.9	-4.0

Values of methylation levels correspond to the average level of methylation of the three CpG sites evaluated for *LINE-1 *and the five CpG sites evaluated for *MLH1 *and *MGMT*. Data were collected using the pyrogram which had the best internal control of conversion.

*: indicates an uncertain result due to the low intensity of the pyrogram. †: indicates an uncertain conversion (5.0 to 7.0% of unconverted cytosines). ‡: indicates a poor conversion (more than 7.0% of unconverted cytosines). Numbers in bold indicate a difference of methylation level outside the -6.0/+6.0% interval. Differences in methylation levels were calculated by subtracting the level of methylation of the cryo-preserved sample from the level of methylation of the FFPE sample.

We chose not to show methylation levels of FFPE DNA extracted with the classical method because most of these DNAs displayed invalid conversion. Nevertheless we noticed that these levels were often higher than the levels of methylation of the same FFPE DNA extracted with the dedicated kit because 40.0% of the cases showed a significantly higher level of methylation (Additional file 2).

**Figure 2:**
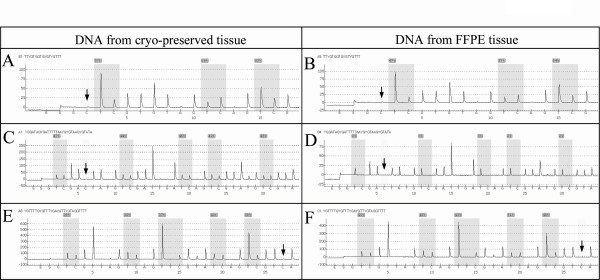
**Pyrograms of the *LINE-1, MLH1 *and *MGMT *methylation markers for different couples of frozen/FFPE DNA**. Pyrograms of *LINE-1 *marker are those obtained for couple n° 10 (**A **and **B**) and for *MLH1 *and *MGMT *markers those for couples n°13 (**C **and **D**) and n°6 (**E **and **F**) respectively. Arrows indicate positions of internal controls of conversion, demonstrating no residual cytosines at the non-CpG sites. Gray areas indicate polymorphisms, between T/C, generated by bisulfite treatment. Level of methylation for a given CpG dinucleotide is reported above it (gray square).

Eight couples (n°10, 11, 13, 18, 19, 25, 28 and 29) presented a difference in *LINE-1 *methylation greater than +6.0% or lower than -6.0% between cryo-preserved and FFPE tissue. For these eight cases, the bisulfite conversion control was successful for all cryo-preserved and FFPE DNAs. Among these eight couples, the level of methylation in five cases was higher for DNA extracted from cryo-preserved tissue than from FFPE tissue and lower in three cases. For the *MLH1 *marker, the differences could be established on 29 couples of DNA. Four couples (1, 9, 13 and 17) displayed a difference in the *MLH1 *methylation level greater than +6.0% or lower than -6.0% with no problem of bisulfite conversion. Among these four couples, three had a level of *MLH1 *methylation that was higher in the DNA extracted from cryo-preserved tissue. With the *MGMT *marker, 15 couples (n° 3, 4, 6, 7, 10, 11, 17, 19, 20, 24, 25, 26, 28, 32 and 37) of DNA presented a difference in the level of *MGMT *methylation greater than +6.0% or lower than -6.0%. Seven of these couples (cases n° 3,7,11,19,26,28,37) showed a problem of bisulfite conversion for one of the two samples. Among the remaining eight couples, the methylation level was higher for DNA extracted from FFPE tissue in six cases (n°6, 10, 17, 20, 24 and 32) and lower for two cases (n° 4 and 25). By combining the analyses of the methylation levels of the three markers (Table 3), no couples of frozen/FFPE DNA presented a difference in the methylation level for the three markers at the same time. Five couples of DNA (n°10,11,19,25 and 28) displayed a difference in methylation levels for both *LINE-1 *and *MGMT *markers, one couple (n°13) for *LINE-1 *and *MLH1 *markers and one couple (n°17) for *MLH1 *and *MGMT*. Examples of pyrograms for couples showing differences in methylation levels are presented in Figure 2.

### Synthetic analysis of bisulfite conversion quality and methylation levels for cryo-preserved and FFPE tissues for the three markers (Table 4)

*For the Line 1 marker*, 29 couples had successful bisulfite conversion. Among these, eight (27.6%) displayed a significant difference in the methylation levels while 21 couples showed no difference in the methylation levels between cryo-preserved and FFPE samples. *For the MLH1 marker*, 26 out of 28 couples for which DNA could be amplified showed satisfactory control of bisulfite conversion. Among these, four (15.4%) displayed a significant difference in the methylation levels while 22 couples showed no difference in the methylation levels between cryo-preserved and FFPE samples. *For the MGMT marker*, 31 couples had successful bisulfite conversion. Among these, eight (25.8%) displayed a significant difference in the methylation levels while 23 couples showed no difference in the methylation levels between cryo-preserved and FFPE samples. All things considered, five couples (n°8,14,15,23,39) displayed both satisfactory control of conversion and no difference in the methylation levels for the three markers.

**Table 4:** Synthetic view of the bisulfite conversion quality and the equivalence of methylation levels

*LINE-1*	1	2	4		6		8	9					14	15		17		20		23	24	26			30	31	32		34	35	36			39	40
*MLH1*				5	6	7	**8**		10	11	12		**14**	**15**	16			20	22	**23**	24	26	28	29			32	33				37	38	**39**	

*MGMT*	1	2		5			**8**	9			12	13	**14**	**15**	16		18		22	**23**				29	30	31		33	34	35	36		38	**39**	40

In each line (according to the corresponding marker) the identities of couples of frozen/FFPE DNAs (designated as couple 1 through couple 40) displaying both successful controls of bisulfite conversion and no differences in methylation levels are listed. Numbers in bold indicates couples displaying both a successful control of bisulfite conversion and no differences in methylation levels for the three markers.

## Discussion

Epigenetic silencing of genes, mostly mediated by aberrant DNA methylation, is a mechanism of gene inactivation in patients with colorectal cancer [2]. Among the loci that can undergo aberrant methylation in colorectal cancer, one subgroup appears to become aberrantly methylated as a specific group [2,4], a phenomenon called the CpG island methylator phenotype (CIMP). Results in the literature on CIMP status as a prognostic factor for colon cancers remain controversial. These conflicting results could result from differences in patient cohorts, samples (the use of frozen or FFPE tissues), analytical techniques, methylation markers (different genes or the same genes but screened at different sites), primer sequences and criteria for CIMP. In our study, we chose to focus on the impact of the pre-analytical phase. A variety of assays to measure DNA methylation have been developed for FFPE and frozen tissues many of which rely on the bisulfite conversion of unmethylated cytosines from tumour tissue into uracils. However, the efficacy of sodium bisulfite treatment and the measurement of methylation levels in FFPE samples in one hand and in frozen samples on the other hand have never really been compared and evaluated.

In this study, we assessed the quality of bisulfite conversion as well as methylation levels for *LINE-1, MLH1 *and *MGMT *markers by pyrosequencing from 40 pairs of FFPE and frozen samples. Pyrosequencing is particularly useful because it provides resolution at the individual nucleotide level and includes a conversion control for each analysis. Assays targeting CpG islands of *LINE-1, MLH1 *and *MGMT *were chosen since aberrant methylation of this retro-transposon and these genes is implicated in colon cancers. *LINE-1 *(long interspersed nucleotide element-1) is a retro-transposable element of DNA that is present in 15% of the human genome. It is a surrogate marker of genome-wide DNA methylation [18,22,23]. It is frequently hypomethylated in human cancers [24,25]. In contrast, the two genes *MLH1 *(the human homolog of the E. coli DNA mismatch repair gene mutL) and *MGMT *(O6-Methylguanine DNA methyltransferase) are hypermethylated in a number of different cancers, including colorectal cancer [26-28].

We first demonstrated that bisulfite-to-bisulfite standard deviations of methylation levels (mean 1.3%) and PCR/pyrosequencing run-to-run standard deviations of methylation levels (mean 0.9%) were low and acceptable. We chose to carry out the assays on frozen tissues to avoid the potential impact of DNA degradation from FFPE tissues. The results obtained for LINE -1 can be extrapolated to any other validated methylated markers. These assays allowed us to consider that our process of bisulfite conversion and PCR/pyrosequencing is reproducible and to establish an arbitrary threshold of 6.0% beyond which the difference in methylation value was not due to variability in the measurements.

To assess the quality of sodium bisulfite conversion and the subsequent PCR/pyrosequencing assays, using the *LINE-1 *marker, we evaluated conversion of 40 FFPE DNAs extracted using a routine method. The results were neither satisfactory nor reliable since only 15 cases showed valid control of conversion after the first analysis and 22 cases (55.0%) after combining the two analyses. We consequently decided to use a dedicated kit to re-extract DNA from FFPE tissues and to extend the assays to the two other markers. All the samples were evaluated using the *LINE-1 *and *MGMT *markers: only 27 cases (67.5%) showed good control of bisulfite conversion with the *LINE-1 *marker and 22 cases (55.0%) with the *MGMT *marker in the first assay. In contrast, with the *MLH1 *marker, in 11 cases amplification failed, possibly because of the length of the *MLH1 *amplicon (181 pb). Among the 29 amplifiable samples, 23 (57.5% of the 40 cases) showed valid control of conversion in the first analysis. Furthermore, the results were not the same in the second analysis. It is thus clear that the extraction phase should not be conducted using a routine process and must comprise a supplementary step of heating to 90°C for 1 h to improve bisulfite conversion. Even with this dedicated method, the rate of satisfactorily converted samples was much lower for FFPE than for frozen samples. In contrast, the proportion of satisfactorily converted samples in the first assay on frozen samples was 95.0% with *LINE-1*, 97.4% with *MLH1 *and 87.2% with *MGMT*. The results were reliable and similar after the second analysis. Furthermore, we did not encounter the problem of failed amplification (due to amplicon length) found with the *MLH1 *marker on FFPE tissues. DNA derived from FFPE is an extremely valuable source of material for retrospective studies, but is often highly degraded. When PCR-based methods are used to study DNA methylation changes, it is necessary to modify the DNA with sodium bisulfite to preserve the DNA methylation information of the original template, and this treatment may further damage the DNA. Fragmentation of FFPE DNA is a real drawback, particularly in DNA methylation studies based on methylation-independent PCR: the design of the primers must be conducted with the highest score, primers have to hybridize out of the CpG islands, and amplicon length has to be limited. It is highly required to respect these constraints when using pyrosequencing which is the only technique allowing a real quantification of methylation.

In our study, FFPE DNAs (n°30 and 36), showed invalid control of conversion with *MLH1 *and good control with *LINE-1 *and *MGMT*. In addition, none of the FFPE DNAs showing invalid control of bisulfite conversion with *LINE-1 *(10 cases) showed invalid conversion with *MLH1*, while in three cases (cases n°3, 21 and 37) the results coincided with those of *MGMT*. As the efficacy of bisulfite conversion appeared to be heterogeneous all along the DNA and variable according to the analysed marker, we think that percentages of bisulfite conversion differ from one cytosine to another due to residual cross-linkings (even with the 90°C heating step) leading to non-reproducible results on FFPE samples with the three markers.

Thus, even when extraction methods dedicated to FFPE tissue were used, problems occurred with bisulfite conversion. The importance of successful bisulfite conversion was underlined by a panel of experts who reported that incomplete conversion of DNA was the major cause of false-positive results in methylation analysis [29]. We chose not to evaluate the results of the methylation levels obtained from FFPE DNA extracted using the classical method because the number of unsatisfactory conversions was too high, but it was clear that poor bisulfite conversion led to overestimation of methylation (Additional file 3: Figure 1). In order to compare the methylation levels of cryo-preserved and FFPE samples (DNA extraction with the dedicated kit), we established an arbitrary threshold (6.0%) beyond which differences in the methylation value were not due to variability in the measurements. We demonstrated that with *LINE-1 *eight pairs (27.6%), with *MLH1*, four pairs (15.4%), and with *MGMT*, eight pairs (25.8%) displayed significant differences in the methylation level. These deviations in methylation levels between matched FFPE and cryo-preserved samples cannot be due to differences in tumour cellularity as we checked that they were slight. In a previous work (data not published) we evaluated the variations in methylation levels induced by variations in tumor cellularity using the *LINE-1 *marker, and we observed a maximal deviation of 3.6% of methylation for a cellularity difference of around 20% from the same sample. This argument is supported by the study published by Irahara et al. [30] who compared average methylation values for *LINE-1 *in macrodissected colon cancers with those for matched Laser Capture Microdissection specimens providing a pure collection of tumor cells. They found no substantial effects of contaminating normal cells on *LINE-1 *methylation.

We found no pairs that showed a difference in methylation level with all three markers and we were unable to establish a trend for the differences in methylation levels in FFPE versus frozen samples. Clearly these deviations in methylation levels differentially impact according to the analyzed marker. For *MLH1 *and *MGMT *markers, a sample is considered as methylated whatever it displayed 20% or 50% of methylation. But for the *LINE-1 *marker, several subgroups of methylation can be distinguished and variations of methylation shown here can be critical for the creation of these subgroups.

Our study is the first to compare the results obtained for DNA extracted from FFPE and frozen tissues to assess the feasibility of DNA methylation analysis using pyrosequencing. Some authors maintain that sodium bisulfite treatment is sufficiently precise and shows good reproducibility on FFPE samples leading to reliable assessment of methylation levels [19,31]. Nevertheless, these authors did not perform comparative studies with frozen material and used techniques such as Methylight (real time PCR), SMART-MSP (Sensitive Melting Analysis after Real Time PCR - methylation-specific polymerase chain reaction), MS-HRM (Methylation Sensitive- High Resolution Melting), without a built-in measure to check the completeness of bisulfite conversion (conversion control). Furthermore, to confirm that FFPE tissue can be effectively used for high-throughput DNA methylation analysis, Ogino et al. [31] used the alternative method of protein expression by immunohistochemistry, which is a surrogate indicator of DNA methylation.

Looking ahead, there is another source of variability that should be investigated: bisulfite conversion methods which vary according to laboratories. This point reinforces the need for standardization in this domain.

## Conclusions

In conclusion, we demonstrated that the use of FFPE tissues induces unsatisfactory and non-reproducible bisulfite conversion leading to unreliable results for methylation levels. In contrast, frozen samples give reproducible results for sodium bisulfite conversion and subsequent pyrosequencing assays have acceptable precision using a single analysis. There is clearly a need to standardize the entire process of DNA methylation analysis from the tissue preservation method to the technology used to quantify methylation. Our results indicate that using FFPE collections to evaluate the prognosis of CIMP colon cancers by bisulfite methods partly contributes to the discrepant data observed in this field. In light of these results, we strongly recommend the use of DNA from cryo-preserved tissue when performing bisulfite conversion to study DNA methylation.

## Abbreviations

*BAX*: Bcl2-associated × protein; CIMP: CpG island methylator phenotype; CpG: Cytosine-phosphate-Guanine; DNA: deoxyribonucleic acid; FFPE: formalin-fixed paraffin embedded; HES: Hematein-Eosin-Safran; *IGFR*: insulin-like growth factor receptor; *LINE-1*: Long Interspersed nucleotide element 1; *MGMT*: O6-Methylguanine DNA methyltransferase; *MLH1*: human MutL homolog 1; MS-HRM: methylation sensitive-high resolution melting; *MSH3*: human MutS homolog 3; MSP: methylation-specific polymerase chain reaction; MSS: microsatellite stable; PCR: polymerase chain reaction; RNase: ribonuclease; dNTP: deoxy-nucleotide triphosphate; SD: standard deviation; SMART-MSP: sensitive melting analysis after real time PCR - methylation-specific polymerase chain reaction; *TGFβRII*: transforming growth factor-beta receptor, type II

## Competing interests

The authors declare that they have no competing interests.

## Authors' contributions

BT, CC, CL and FP instigated the study. EC and LM participated in the study design. BT carried out the DNA extractions and the pyrosequencing experiments. BT and FP drafted the manuscript. CC, CL and JF were involved in revising it critically. All authors read and approved the final manuscript.

## Pre-publication history

The pre-publication history for this paper can be accessed here:

http://www.biomedcentral.com/1471-2407/12/12/prepub

## Supplementary Material

Additional file 1 **This file is in Microsoft Word 97-2003 format**. It includes a detailed description of the protocol for the extraction method dedicated to FFPE tissue and of the protocol for the DNA bisulfite conversion Click here for file

Additional file 2 **This file is in Microsoft Word 97-2003 format**. This file contains the *LINE-1 *primer sequences (Supplementary table 2), the dispensation orders of nucleotides for the three markers (Supplementary table 2) and data on the *LINE-1 *methylation levels obtained from FFPE DNA extracted by the classical method (Supplementary table 3). Click here for file

Additional file 3 **This file is in JPEG format**. This figure is entitled: Pyrograms of the *LINE-1 *methylation marker for FFPE DNA n°2 extracted with the classical method. It groups the two pyrograms obtained from the two *LINE-1 *analyses of the FFPE DNA n°2 extracted with the classical method. Click here for file
