# Supplementary material for: Why do results conflict regarding the prognostic value of the methylation status in colon cancers? the role of the preservation method
Source: BMC Cancer. 2012 Jan 13;12:12. doi: 10.1186/1471-2407-12-12 (PMC3293017; doi:10.1186/1471-2407-12-12)
Supplement: Additional file 1 — This file is in Microsoft Word 97-2003 format. It includes a detailed description of the protocol for the extraction method dedicated to FFPE tissue and of the protocol for the DNA bisulfite conversion [file 1471-2407-12-12-S1.DOC]

**Supplementary materials and methods:**

**Dedicated Method to FFPE tissue: QIAamp® DNA FFPE Tissue kit (QIAGEN®)**

For each sample, ten 15-µm-thick tissue sections were transferred into a 1.5 ml tube and 1 ml of toluene was added to dissolve the paraffin. The tubes were vigorously vortexed for 10 seconds, then centrifuged at 20,000 g for 2 minutes and the supernatants were then removed. One ml of absolute ethanol (96-100%) was added to the pellets. The mixes were then vortexed, and centrifuged at 20,000 g for 2 minutes. The supernatants were removed. The tubes were opened and incubated at room temperature until all residual ethanol was evaporated. Lysis was performed by adding 180 µl of lysis buffer ATL plus 20 µl of proteinase K and by incubating at 56°C for 1 hour. The samples were heated to 90°C for one hour to reverse formaldehyde-induced DNA modifications. The tubes were then briefly centrifuged and all of the lysates were transferred into QIAamp MinElute columns (located in 2 ml collection tubes). The columns were centrifuged at 6,000 g for 1 minute and flow-throughs were discarded. Then residual contaminants were washed away by two successive steps using two different buffers (firstly AW1, secondly AW2): 500 µl of buffer was added and the columns were centrifuged at 6,000 g for 1 minute, flow-throughs were discarded. To completely dry the membrane, the columns were centrifuged at 20,000 g for 3 minutes. Columns were after placed in clean 1.5 ml microcentrifuge tubes. Then 100 µL of buffer ATE (elution buffer) was dispensed onto the center of membranes and the columns were incubated at room temperature for 1 minute. Finally, pure DNA was eluted by centrifugation at 20,000 g for 1 minute.

**DNA bisulfite conversion - EpiTect Bisulfite kit (QIAGEN®)**

For bisulfite conversion, 500 nano-grams of genomic DNA of each sample were used and bisulfite treatment was performed according to the manufacturer’s protocol. First the bisulfite reaction was prepared by mixing DNA solution (1 to 20 µl – depending on the concentration of DNA solution) with RNase-free water (variable volume – the combined volume of DNA solution and RNase-free water must total 20 µl), 85 µl of Bisulfite Mix and 35 µl of DNA Protect Buffer. To perform the bisulfite DNA conversion, a thermal cycling incubation was applied as follows: 5 minutes to 95°C – 25 minutes to 60°C - 5 minutes to 95°C – 85 minutes to 60°C - 5 minutes to 95°C – 175 minutes to 60°C. Once the conversion was complete, the mixtures were transferred to 1.5 ml microcentrifuge tubes to which were added 310 µl of Buffer BL containing 10 µg/ml carrier RNA. The solutions were vortexed and briefly centrifuged. Then, 250 µl of absolute ethanol (95-100%) were added and the content of the tubes was mixed by pulse vortexing for 15 seconds, and briefly centrifuged. All of the mixtures were transferred into EpiTect spin columns (located in 2 ml collection tubes). The columns were centrifuged at 20,000 g for 1 minute and flow-throughs were discarded. 500 µl of Buffer BW (wash buffer) was added and the columns were centrifuged at 20,000 g for 1 minute and the flow-throughs were discarded. The columns were incubated with 500 µl of Buffer BD (desulfonation buffer) at room temperature for 15 minutes. They were then centrifuged at 20,000 g for 1 minute and flow-throughs were discarded. Then two identical steps of washing were successively carried out: 500 µl of Buffer BW (wash buffer) was added and the columns were centrifuged at 20,000 g for 1 minute, and the flow-throughs were discarded. To remove any residual liquid, the columns were centrifuged at 20,000 g for 1 minute then placed with open lids in a heating block at 56°C for 5 minutes. The columns were then placed in clean 1.5 ml microcentrifuge tubes. Purified converted DNA was eluted by dispensing 30 µl of Buffer EB onto the center of each membrane and centrifuging at 15,000 g for 1 minute.
