# Supplementary material for: Why do results conflict regarding the prognostic value of the methylation status in colon cancers? the role of the preservation method
Source: BMC Cancer. 2012 Jan 13;12:12. doi: 10.1186/1471-2407-12-12 (PMC3293017; doi:10.1186/1471-2407-12-12)
Supplement: Additional file 2 — This file is in Microsoft Word 97-2003 format. This file contains the LINE-1 primer sequences (Supplementary table 2), the dispensation orders of nucleotides for the three markers (Supplementary table 2) and data on the LINE-1 methylation levels obtained from FFPE DNA extracted by the classical method (Supplementary table 3). [file 1471-2407-12-12-S2.DOC]

| **Primer identity** | **Sequence 5’®3’** |
| --- | --- |
| Forward primer | TTTTGAGTTAGGTGTGGGATATA |
| Reverse primer | Biotine-AAACCCAAAAAATCAAAAAATTCCCTTTCC |
| Sequencing primer | AGGTGTGGGATATAGT |

**Supplementary table 1 - Nucleotide sequences of LINE-1 primers**

| **Marker** | **Dispensation order (5’®3’)** |
| --- | --- |
| LINE-1 | GCTCGTGTAGTCAGTCG |
| MLH1 | GTCGACTATGTCGATTGATCAGTCGTATGTCGTA |
| MGMT | GTCGTTAGTCAGTTCGTATCAGTCGTCA |

**Supplementary table 2 - Dispensation orders of nucleotides for LINE-1, MLH1 and MGMT markers**

| Identity | Levels of LINE-1 methylation (% of methylation) | | Differences of methylation |
| --- | --- | --- | --- |
|  | FFPE DNA extracted with the classical method | FFPE DNA extracted with the dedicated kit |  |
| 1 | 75.0 † | 66.3 | **8.7** |
| 2 | 64.7 | 67.2 | **-2.5** |
| 3 | 66.4 | 64.8 † | **1.6** |
| 4 | 59.6 ‡ | 52.3 | **7.4** |
| 5 | 68.5 | 64.2 † | **4.3** |
| 6 | 56.1 | 49.2 | **6.8** |
| 7 | 64.3 ‡ | 56.6 † | **7.8** |
| 8 | 66.5 | 46.5 | **20.0** |
| 9 | 58.0 | 61.2 | **-3.1** |
| 10 | 77.4 ‡ | 54.1 | **23.3** |
| 11 | 54.5 ‡ | 50.1 | **4.4** |
| 12 | 76.2 ‡ | 62.2 † | **13.9** |
| 13 | 60.7 † | 62.5 | **-1.9** |
| 14 | 59.5 | 60.8 | **-1.3** |
| 15 | 64.3 † | 63.2 | **1.1** |
| 16 | 45.9 | 47.5 † | **-1.6** |
| 17 | 65.1 ‡ | 55.6 | **9.4** |
| 18 | 53.4 | 53.5 | **-0.1** |
| 19 | 60.4 † | 54.4 | **6.0** |
| 20 | 60.8 | 57.6 | **3.3** |
| 21 | 77.0 † | 66.1 † | **10.9** |
| 22 | 50.9 | 49.1 † | **1.8** |
| 23 | 63.0 | 59.4 | **3.7** |
| 24 | 63.1 | 58.0 | **5.1** |
| 25 | 63.3 | 71.0 | **-7.7** |
| 26 | 56.0 † | 52.2 | **3.8** |
| 27 | 51.6 | 44.3 | **7.3** |
| 28 | 55.7 | 57.4 | **-1.7** |
| 29 | 69.1 † | 57.3 | **11.8** |
| 30 | 57.1 | 56.7 | **0.4** |
| 31 | 72.9 ‡ | 62.9 | **10.0** |
| 32 | 64.9 | 62.0 | **3.0** |
| 33 | 57.9 | 62.4 † | **-4.5** |
| 34 | 64.5 † | 61.7 | **2.8** |
| 35 | 83.6 † | 53.3 | **30.3** |
| 36 | 44.7 | 41.1 | **3.5** |
| 37 | 62.1 | 62.1 † | **0.0** |
| 38 | 66.0 | 58.5 † | **7.5** |
| 39 | 58.8 † | 53.2 | **5.6** |
| 40 | 45.6 † | 34.7 | **10.9** |

**Supplementary table 3 - Differences in LINE-1 methylation levels between FFPE DNA extracted with the classical method and the dedicated kit**

Values of methylation levels correspond to the average level of methylation of the three CpG sites. Data were collected using the pyrogram which had the best internal control of conversion. *: indicates an uncertain result due to the low intensity of the pyrogram. †: indicates an uncertain conversion (5.0 to 7.0% of unconverted cytosines). ‡: indicates a poor conversion (more than 7.0% of unconverted cytosines). Grey indicates a difference of methylation level outside the -6.0/+6.0% interval. Differences in methylation levels were calculated by subtracting the level of methylation of the cryo-preserved sample from the level of methylation of the FFPE sample.
