# Supplementary figures and images for: Why do results conflict regarding the prognostic value of the methylation status in colon cancers? the role of the preservation method
Source: BMC Cancer. 2012 Jan 13;12:12. doi: 10.1186/1471-2407-12-12 (PMC3293017; doi:10.1186/1471-2407-12-12)

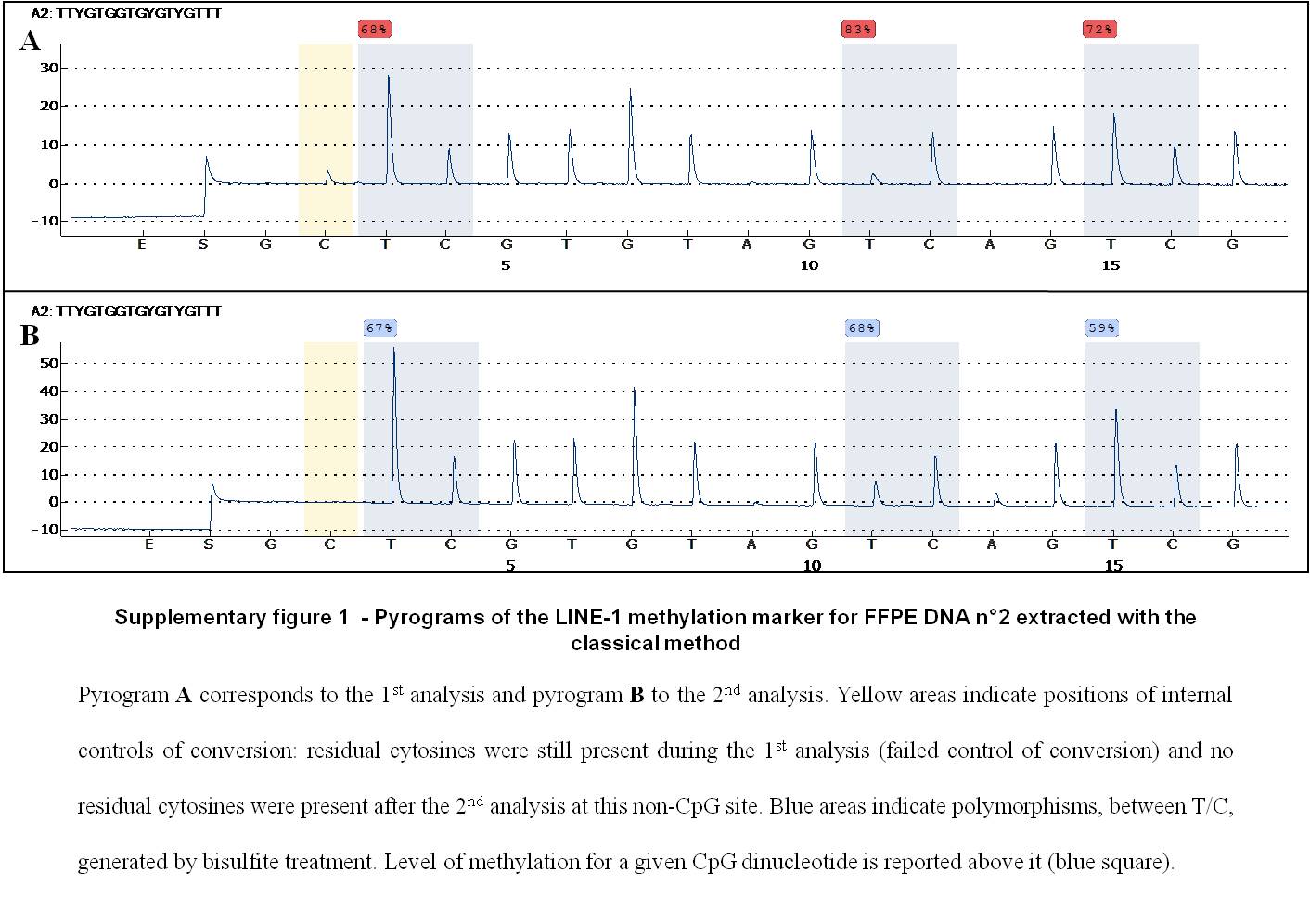

Supplement: Additional file 3 — This file is in JPEG format. This figure is entitled: Pyrograms of the LINE-1 methylation marker for FFPE DNA n°2 extracted with the classical method. It groups the two pyrograms obtained from the two LINE-1 analyses of the FFPE DNA n°2 extracted with the classical method. [file 1471-2407-12-12-S3.JPEG]
